# The Prophylactic Effects of Metoprolol, Diltiazem, and Pilocarpine on Hypoglycemia-Induced Prolongation of QT Interval

**DOI:** 10.7759/cureus.14058

**Published:** 2021-03-23

**Authors:** Eylem Cagiltay, Sjaak Pouwels, Oytun Erbas, Dilek Taskiran, Sevgi Kalkanli Tas, Ismael Aslan

**Affiliations:** 1 Department of Physiology, Istanbul Bilim University School of Medicine, Istanbul, TUR; 2 Intensive Care Medicine, Elisabeth-Tweesteden Hospital, Tilburg, NLD; 3 Department of Physiology, Ege University School of Medicine, Izmir, TUR; 4 Department of Immunology, University of Health Sciences, Faculty of Medicine, Istanbul, TUR; 5 Department of Pharmacy, University of Health Sciences, Institute of Health Sciences, Istanbul, TUR

**Keywords:** hypoglycemia, qtc interval, metoprolol, pilocarpine, diltiazem

## Abstract

Background

Insulin-induced hypoglycemia has been demonstrated to prolong the corrected QT (QTc) interval. Prolongation of the QTc interval, especially in diabetic patients using insulin, can cause fatal ventricular arrhythmias. The aim of this study was to evaluate the effects of metoprolol, diltiazem, and pilocarpine on hypoglycemia-induced QTc prolongation.

Methods

Thirty male rats were randomly distributed into the following five groups: Group 1 (1 mL/kg saline, n=6), Group 2 (40 U/kg crystalline insulin + saline, n=6), Group 3 (40 U/kg crystalline insulin + 1 mg/kg metoprolol, n=6), Group 4 (40 U/kg crystalline insulin + 0.8 mg/kg pilocarpine, n=6), and Group 5 (40 U/kg crystalline insulin + 2 mg/kg diltiazem, n=6). Three hours after insulin injection, the blood glucose level was measured in all groups. Blood glucose <40 mg/dl was defined as hypoglycemia. Electrocardiograms (ECG) were taken in lead I (DI), and QTc was calculated by using Bazett’s formula.

Results

Group 2 (insulin + saline) showed that it had a significantly prolonged QTc interval as compared to the control group (p<0.0001). However, treatments of the rats with metoprolol, pilocarpine, and diltiazem significantly prevented prolongation of the QTc interval as compared to the insulin + saline group (p<0.005, p<0.005, and p<0.01, respectively).

Conclusion

The findings of the present study demonstrated the efficacy of metoprolol, pilocarpine, and diltiazem in the prevention of hypoglycemia-induced QTc prolongation in male rats.

## Introduction

Hypoglycemia may play a critical role in the sudden death of young people with diabetes mellitus. Several clinical and experimental studies have confirmed the pro-arrhythmic effects of hypoglycemia in type 1 and type 2 diabetes patients [1-2]. Hypoglycemia may delay ventricular repolarization, prolong the corrected QT (QTc) interval, and stimulate severe ventricular cardiac arrhythmias, predisposing the patient to sudden arrhythmic death [3]. The sympathoadrenal activation, hypokalemia, and Ca2+ overload during hypoglycemia are the major mechanisms responsible for the QTc prolongation [4]. Preexistent cardiovascular diseases and diabetes are also related to long QT syndrome (LQTS) and Ca2+ excess, and, therefore, increase the probability of proarrhythmic effects during hypoglycemia [5-10].

Metoprolol is a member of selective beta-blockers and class II anti-arrhythmic agents [11-13]. Metoprolol decreases the heart rate and is effective for the treatment of supraventricular tachycardia and ventricular tachycardia. Metoprolol is used to prevent arrhythmic events in patients with certain forms of congenital long QT syndrome [12]. Diltiazem, a calcium channel blocker, is a member of class IV anti-arrhythmic agents. Its ability to inhibit atrioventricular (AV) nodal conduction and prolong AV nodal refractoriness is employed in the management of supraventricular tachyarrhythmias [12]. Pilocarpine, a muscarinic acetylcholine receptor (mAChR) agonist, is extensively used for the treatment of xerostomia and glaucoma. Its modulatory effects have been previously demonstrated on the cellular electrical properties of the hearts, likely by stimulating a K+ current mediated by M3 receptors [14].

In the present study, we aimed to investigate whether these agents (metoprolol, diltiazem, and pilocarpine) have beneficial effects on the hypoglycemia-induced prolongation of QTc interval in an experimental rat model. This paper has been presented as a poster at the 22nd European Congress of Endocrinology (eECE 2020, online edition) on September 5-9, 2020 [15].

## Materials and methods

Animals

Thirty adult male Sprague Dawley (220-240 g) rats were included in the study. Animals were fed ad libitum and housed in pairs in steel cages having a temperature-controlled environment (22±2 °C) with 12-h light/dark cycles.

The experiments performed in this study have been carried out according to the rules in the Guide for the Care and Use of Laboratory Animals adopted by the National Institutes of Health (U.S.A). Having received Animal Ethics Committee’s consent (Ethical number 2013 HADYEK-032), the rats used in the experiment were obtained from the Experimental Animal Laboratory of Science University (Istanbul Bilim University, Istanbul, Turkey). All experiments and procedures were conducted in accordance with the Animal Research: Reporting of In Vivo Experiments (ARRIVE) guidelines and an ARRIVE checklist was provided with this manuscript.

Experimental design

Animals were randomly distributed into five groups consisting of six rats in each. Group 1 was used as the control and received 1 mL/kg saline intraperitoneally (i.p.); 40 U/kg crystalline insulin and saline were administered i.p. to Group 2; 40 U/kg crystalline insulin and 1 mg/kg metoprolol were administered i.p. to Group 3; 40 U/kg crystalline insulin and 0.8 mg/kg pilocarpine were administered i.p. to Group 4; 40 U/kg crystalline insulin and 2 mg/kg diltiazem were administered i.p. to Group 5. Three hours after insulin injection, blood sugar was measured in all study groups of rats. Blood glucose <40 mg/dl was defined as hypoglycemia.

All rats were euthanized at the end of the experimental period. First, high-dose anesthesia (200 mg/kg ketamine hydrochloride) was applied. Then cervical dislocation was performed by sacrification.

Anaesthesia and ECG recordings

Electrocardiograms (ECG) were taken in lead I (DI) (Biopac MP 150, BIOPAC Systems, Inc., CA) under anesthesia. Rats were anesthetized by a combination of ketamine hydrochloride at a dose of 40 mg/kg (Alfamine, Ege Vet, Alfasan International B.V.Woerden, Holland) and 4 mg/kg of xylazine hydrochloride (Alfazyne, Ege Vet, Alfasan International B.V. Woerden, Holland), which was administered i.p. ECGs were recorded in the prone position. Ketamine has fewer effects on glucose and insulin metabolism in animals, especially in rats. Therefore, we chose ketamine as the sedative agent [3-4,6].

Electrodes consisted of 26-gauge needles placed subcutaneously for 1 cm. Standard limb leads were constructed from electrodes placed at the paws [16]. Data were evaluated using Biopac Student Lab Pro software, version 3.6.7 (BIOPAC Systems, Inc., CA), and the parameters were as follows: QT interval, T-wave duration, and heart rate [16]. Bazett’s formula was used for the calculation of QTc [16].

Statistical analysis

Data management and analysis were performed using the Statistical Package for the Social Sciences (SPSS) version 24 for Mac (IBM Corporation, Armonk, NY). Due to the exploratory nature of this study, no power and sample size calculations were done. Continuous variables were presented as mean ± standard error of the mean (SEM). Categorical variables were presented as frequency with percentages. The chi-square test was used to assess the differences between categorical variables between the groups. The Shapiro-Wilk test was used to test each variable for normality. Student’s t-test for independent groups, the Mann-Whitney U test, or analysis of variance (ANOVA) was used, depending on the normality or non-normality of the data distribution. The investigators who measured the QT intervals and performed the data analysis were blinded to the treatment assignments. P-values of ≤ 0.05 were considered statistically significant.

Patient and public involvement

Hypoglycemia may play a critical role in the sudden death of young people with diabetes mellitus due to the prolongation of the QT interval [1-2]. Therefore, it is of interest to investigate the effects of medication on hypoglycemia-induced QT interval enlargement and delay of ventricular repolarization. Since this is an animal study, there was no active involvement of patients in the development of this study. After publication, the results of this study will be disseminated across several patient groups in the fields of cardiology and diabetology.

## Results

**Figure 1:**
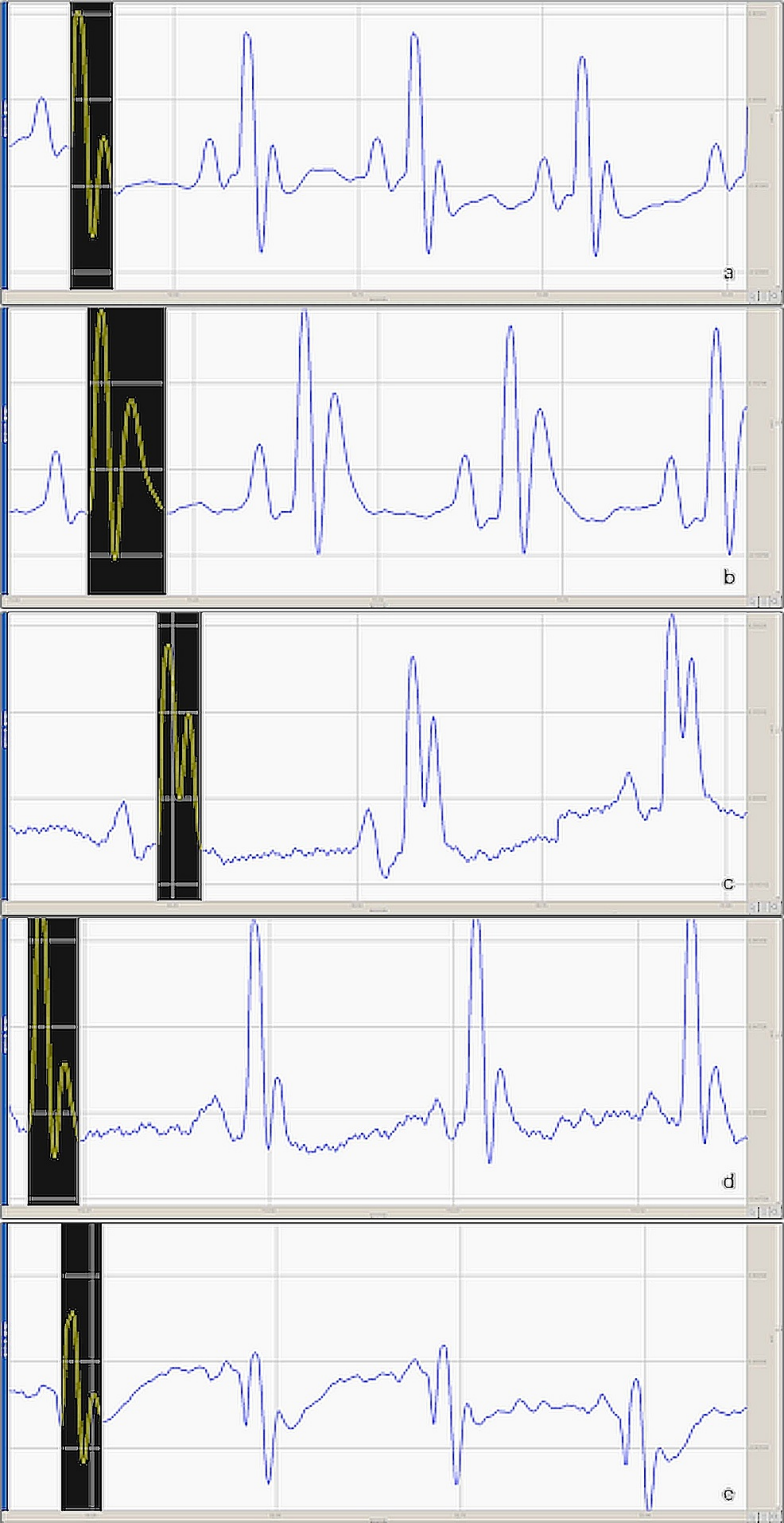
Electrocardiogram (ECG) alterations in experimental groups QT intervals are shown with a black band for each group. (a) Control group, (b) Insulin and saline group, (c) Insulin and metoprolol group, (d) Insulin and pilocarpine group, (e) Insulin and diltiazem group

Figure 1 demonstrates the ECGs of all groups, and Table 1 presents the effect of metoprolol, diltiazem, and pilocarpine on ECG parameters.

**Table 1:** Electrocardiogram (ECG) parameters of normoglycemic and hypoglycemic rats that received saline, metoprolol, pilocarpine, and diltiazem Data are expressed as mean ± SEM. * p < 0.0001 vs. control; ** p < 0.00001 vs. control; † p < 0.01 vs. insulin + saline; # p < 0.005 vs. insulin + saline; ## p < 0.0005 vs. insulin + saline

	QTc (msec)	BPM (Beat per minute)	T duration (msec)	QT duration (msec)
Control (saline)	124.7 ± 1.74	264.33 ± 8.86	28.42 ± 0.61	61.33 ± 0.33
Insulin + saline	143.8 ± 4.31 *	288.16 ± 10.67	43.57 ± 0.70 **	71.33 ± 0.88 **
Insulin + metoprolol	121.6 ± 1.83 #	201.33 ± 8.65 ##	31.5 ± 1.25 ##	61.0 ± 1.46 ##
Insulin + pilocarpine	116.6 ± 0.98 #	232.0 ± 11.33 ##	29.0 ± 0.96 ##	58.0 ± 1.09 ##
Insulin + diltiazem	129.0 ± 0.40 †	247.0 ± 6.58 #	30.33 ± 1.11 ##	60.66 ± 0.42 ##

Group 2 (insulin + saline) was found to have a significantly prolonged QTc interval (143.8 ± 4.31 msec) as compared to the control group (124.7 ± 1.74 msec) (p<0.0001). However, treatments of the rats with metoprolol, pilocarpine, and diltiazem significantly prevented the prolongation of the QTc interval as compared to the insulin + saline group (p<0.005, p<0.005, and p<0.01, respectively). Besides, there were statistically significant differences between Group 2 (insulin + saline) and other treatment groups by means of QT and T durations. In Group 2, QT and T durations were significantly longer than the control group (p<0.00001). However, Group 3 (insulin + metoprolol), Group 4 (insulin + pilocarpine), and Group 5 (insulin + diltiazem) revealed significantly shorter QT and T durations than that of the rats received insulin + saline (p<0.0005). Heart rate (BPM) was also significantly decreased in metoprolol, pilocarpine, and diltiazem treated groups as compared to the insulin + saline group (p<0.0005, p<0.0005, and p<0.005, respectively).

## Discussion

In the present experimental study, we demonstrated the potential prophylactic and therapeutic effects of pilocarpine, metoprolol, and diltiazem in the hypoglycemia-induced QT prolongation.

Hypoglycemia is the most frequent adverse effect of intensive insulin therapy in type 1 and type 2 diabetes [17]. Although intensive glucose control reduces the risk of the development of complications of diabetes, it also invariably increases the risk for hypoglycemia [18]. In recent large randomized trials, such as Action in Diabetes and Vascular Disease: Preterax and Diamicron Modified Release Controlled Evaluation (ADVANCE), Veterans Affairs Diabetes Trial (VADT), and Action to Control Cardiovascular Risk in Diabetes (ACCORD), it has been shown that intensive glucose control has no benefit and is associated with increased all-cause mortality [19]. The ACCORD study is the first large controlled clinical trial that raises the fundamental question of whether hypoglycemia induces ventricular tachycardia and fibrillation and increases the risk of sudden death [20].

Dead in bed syndrome is a term used for describing the sudden death of patients with type 1 diabetes [17]. Definitive scientific evidence is lacking for the cause and mechanisms leading to this very rare phenomenon. Possible predisposing factors for this syndrome may be both nocturnal hypoglycemia and cardiac autonomic dysfunction [21-22].

Each of these factors may delay ventricular repolarization, prolong the QT interval, and induce cardiac arrhythmias, predisposing the individual to sudden arrhythmic death [3]. There is considerable evidence that experimental and spontaneous insulin-induced hypoglycemia affects cardiac repolarization. Several studies have demonstrated the hypoglycemia-induced fatal cardiac ventricular arrhythmias and QT prolongation [22-25]. This altered repolarization is notable on the ECG as a flattened T wave and a prolonged QTc interval [24].

Several hypotheses have been proposed to explain the development of the malignant arrhythmogenic effect of hypoglycemia such as sympatho-adrenergic activation, specific changes in K+ currents, and Ca2+ loading. It has been shown that insulin-induced hypoglycemia has K+ channel blocking effects predominantly affecting the delayed rectifier repolarizing current (IK), which has a major role in the electrical activity of cardiac ventricular myocytes [26].

Hypoglycemia is also associated with increased sympatho-adrenergic activation and catecholamine release, which have profound effects on myocardial workload, myocardial contractility, and cardiac output [27]. The increased catecholamine release during hypoglycemia raises intracellular Ca2+, thereby increasing the risk of QTc prolongation, ventricular tachycardia, and sudden death. The sympathetic response and hypoglycemia-induced hypokalemia may cause additional QT prolongation and Ca2+ excess [24, 28-29]. Selective beta-blockers, such as propranolol and nadolol, are frequently used in patients with an increased risk of prolonged QT during hypoglycemia [24,28-29].

Parasympathetic activation during insulin-induced hypoglycemia may be also anti-arrhythmic. Parasympathetic activation may lower the heart rate and decrease the amount of Ca2+ loading from the stimulation of the sympathetic nervous system. Bradycardia has been demonstrated during hypoglycemic episodes in patients. Anti-arrhythmic effects of pilocarpine [15] and choline [24,28-29] have been shown previously in experimental studies. The effect may be possibly due to the lessening of Ca2+ overload and delayed after depolarization.

Due to the reasons mentioned above, we selected metoprolol, pilocarpine, and diltiazem in our study to evaluate whether they have a prophylactic effect on hypoglycemia-induced QT prolongation. In the present study, first of all, we validated QTc prolongation in the first group of hypoglycemic rats that received only saline. The QTc intervals in the second, third, and fourth groups of hypoglycemic rats that received metoprolol, pilocarpine, and diltiazem were significantly shorter than in hypoglycemic rats that received saline. However, we have to take into account that feeding the animals ad libitum could influence the QT interval. These findings clearly suggest the beneficial effects of these drugs against the pro-arrhythmic effects of hypoglycemia.

## Conclusions

Better knowledge of the probable cardiotoxic effects of insulin-induced hypoglycemia is a crucial approach in lessening cardiotoxicity. The initiation of prophylactic therapy with pilocarpine, metoprolol, or diltiazem may be useful in preventing the pro-arrhythmic effects of insulin-induced hypoglycemia.
